# Effect of *α*-Allocryptopine on Delayed Afterdepolarizations and Triggered Activities in Mice Cardiomyocytes Treated with Isoproterenol

**DOI:** 10.1155/2015/634172

**Published:** 2015-10-19

**Authors:** Bin Xu, Yicheng Fu, Li Liu, Kun Lin, Xiaojing Zhao, Yu Zhang, Xi Chen, Zhongqi Cai, Yun Huang, Yang Li

**Affiliations:** ^1^Department of Cardiology, General Hospital of Chinese People's Liberation Army, Beijing 100853, China; ^2^Department of Gerontology, Union Hospital, Tongji Medical College, Huazhong University of Science and Technology, Wuhan 430030, China

## Abstract

*Objective*. To investigate the effect of *α*-allocryptopine (ALL) on delayed afterdepolarization (DAD) incidence and triggered activity (TA) in mice administered isoproterenol (ISO). *Methods*. Mouse ventricular myocytes were isolated. And the cellular electrophysiological properties of ventricular myocytes were investigated. *Results*. We found that the incidences of DADs and TA in mouse myocytes were increased by ISO treatment. In sharp contrast, triggered arrhythmia events were rarely observed in myocytes with 10 *μ*M ALL treatment. Transient inward current (*I*_ti_) was reduced significantly with ALL treatment, which contributed to DAD-related triggered arrhythmia. Compared to Iso-treated group, the L-type calcium current (*I*_Ca,L_) densities were decreased after exposure to ALL, along with slower activation, quicker inactivation, and longer time constant of recovery from inactivation kinetics. *Conclusion*. There is less triggered arrhythmia events in ventricular myocytes treated with ALL. This effect may be associated with the inhibition of *I*_ti_ and *I*_Ca,L_.

## 1. Introduction

It is widely believed that the incidences of delayed afterdepolarizations (DADs) and triggered activities (TAs) have dramatically increased in heart failure patients [1, 2]. DAD-mediated TA is believed to play an important role in abnormal autorhythmicity, which causes the majority of sudden cardiac death during nonischemic heart failure by contributing to intracellular Ca^2+^ overload. DAD-mediated TA leads to activation of sodium-calcium exchanger (NCX) that extrudes Ca^2+^ in exchange for Na^+^ and generates a net inward current (*I*_ti_) [3, 4]. The larger *I*_Ca,L_ plays a vital role in producing *I*_ti_. *I*_ti_ induces DADs that may reach threshold and trigger premature beats [5]. Currently, there is no effective strategy for the treatment of triggered arrhythmias. Available clinical antiarrhythmic drugs have a narrow therapeutic index, which identifies the need for researchers to explore the safety profile and effectiveness of alternative drugs.

*α*-Allocryptopine (ALL), a derivative of tetrahydropalmatine, is extracted from* Corydalis decumbens* (Thunb.) Pers. Papaveraceae [6]. Previous studies have indicated that ALL has antiarrhythmic effects in various animal models, which could be accounted for by the electrophysiological effects of ALL in prolonging the action potential duration [7]. However, it is not clear whether ALL reduces DADs and TAs in ventricular myocardium to resist the development of triggered arrhythmias. The present study aimed to characterize the action and mechanism of ALL on DADs and TAs in mouse ventricular myocytes by using the whole cell patch-clamp technique.

## 2. Materials and Methods

All experimental procedures and protocols were carried out according to the Chinese law on animal experimentation and approved by the Animal Experimental Committee of Chinese PLA General Hospital. This study conforms to the Guide for the Care and Use of Laboratory Animals published by the US National Institutes of Health (NIH Publication number 85-23, revised 1996).

### 2.1. Alpha-Allocryptopine Preparation and Treatment

**Figure 1:**
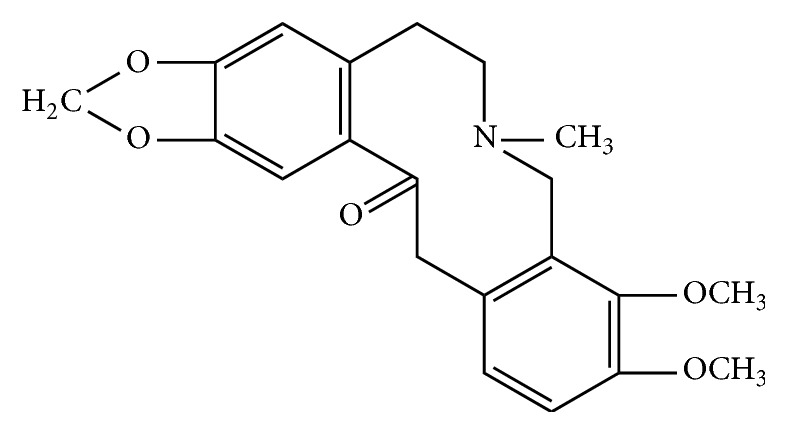
Chemical formula of ALL, an alkaloid extracted from* Corydalis decumbens* (Thunb.) Pers. Papaveraceae.

Alpha-allocryptopine (ALL, formula is shown in Figure 1) was supplied by the Pharmaceutical Department of Lanzhou University (molecular weight: 365), melting point 168°C, as a white crystal powder, and 99.0% purity. ALL was dissolved in dimethyl sulfoxide (DMSO) to obtain a stock solution of 1.0 M. The effect of ALL was investigated at a maximum concentration of 0.5 mM. Based on the highest employed drug concentrations, DMSO was added to the media to produce a final concentration of 0.1%. The drug stock solution was added to the culture media or bath solution to produce the final concentration as reported in Section 3.

### 2.2. Patch-Clamp Experiments

Mouse cardiac myocytes were isolated using an established enzymatic digestion protocol. Cell pellets were resuspended and plated on laminin-coated 35 mm dishes. Only quiescent, Ca^2+^-tolerant, and rod-shaped cells were used. Transmembrane action potentials and currents were recorded in whole cell configuration as previously described using a MultiClamp 700B amplifier (Axon Instruments) [7]. Correction for liquid junction potentials (which averaged −10 mV) was applied only for resting potential and reversal potential values.

Action potentials and current were recorded using the whole cell patch-clamp technique with a MultiClamp 700B amplifier (Axon Instruments). Data were sampled at 10 kHz and subsequently filtered at 5 kHz for analysis (Digidata 1440A, Axon Instruments). Patch pipettes were pulled from borosilicate glass on a P-97 horizontal puller (Sutter Instruments). The resistance electrodes of 2 MΩ~5.5 MΩ were used to record action potentials and currents. A routine series resistance compensation was performed for values >80% to minimize voltage clamp errors. Thus, the uncompensated *R*_series_ was < 2 MΩ. The membrane capacitance was measured on each of the cells and was compensated by approximately 80%~90% of their initial value.

### 2.3. Stimulation Parameters of Cardiac Myocytes

Myocytes were electrically stimulated for 3 ms with a 1.5 nA depolarizing pulse. Action potentials were induced in cardiac myocytes by 30 trains of suprathreshold current pulses at frequencies of 1.0, 2.0, 3.0, 4.0, and 5.0 Hz while the cells were under current clamp. DAD is defined as a depolarization >5 mV for >10 ms occurring during diastole (phase 4) immediately after an action potential. TA is defined as a spontaneous action potential arising from DADs.

To record transient inward current (*I*_ti_), serial voltage steps were preceded by 20 conditioning pulses with each pulse lasting 150 ms from −80 mV to +50 mV at intervals of 100 ms. Following conditioning, serial voltage steps were applied for 2 s from −100 mV to +30 mV at 10 mV increments. Successive trains were 6 seconds apart. *I*_ti_ amplitude was measured as the difference between the peak and the base of the transient current with the first peak taken for analysis.

Using an extracellular solution with 0.05 mM TTX to inhibit Na^+^ current and 5.0 mM CsCl to inhibit K^+^ current, L-type calcium current (*I*_Ca,L_) was recorded with 200 ms depolarizing pulses from a holding potential of −40 mV, with 10 mV steps from −40 mV to +60 mV. Current-voltage (*I*-*V*) curves were obtained with 10 mV voltage steps (−40 mV to +60 mV) from a holding potential of −40 mV.

### 2.4. Statistical Analysis

Statistical analyses were performed using SPSS version 17.0. One-way ANOVA with a Bonferroni post hoc test or Student's *t*-test was used. Chi-square tests (Fisher exact tests) were used to compare differences in the occurrence of DADs and TAs. Data were expressed as mean ± SEM. *p* < 0.05 was considered statistically significant.

## 3. Results

### 3.1. DADs and TA Incidences in Mouse Ventricular Myocytes of Mice with ALL Treatment

**Figure 2:**
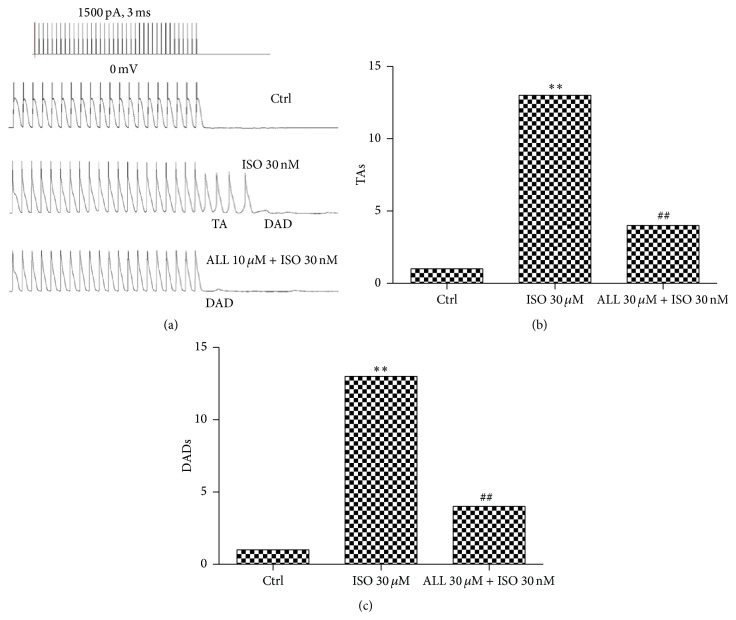
Effects of ALL on DADs and TA in mouse ventricular myocytes with 5.0 Hz stimulation. (a) The events of DADs or TAs induced with ISO were decreased after exposure to 10 *μ*M ALL. The percent of DADs and TA events is shown in (b and c). ^*∗∗*^ *p* < 0.01, compared to control myocytes; ^##^ *p* < 0.01, compared to myocytes with ISO treatment.

Under fast frequency pacing pulse (5.0 Hz), DADs were elicited in 20% (5/25) of cardiac myocytes following 30 nM ISO treatment (Figures 2(a) and 2(b)). The occurrence of DADs was significantly decreased in cardiac myocytes treated with 30 nM ISO treatment when administered 10 nM ALL (9%, 3/25, *p* < 0.01, Figures 2(a) and 2(b)). Only 4% of untreated myocytes had DADs (1/25, Figures 2(a) and 2(b)). Twelve percent of TA events were observed in cells treated with ISO. In myocytes administered ISO and then treated with 10 *μ*M ALL, only 4% had TA events (*p* < 0.01, Figures 2(a) and 2(c)).

### 3.2. Frequency Characteristics of ALL on DADs and TA Incidences

**Figure 3:**
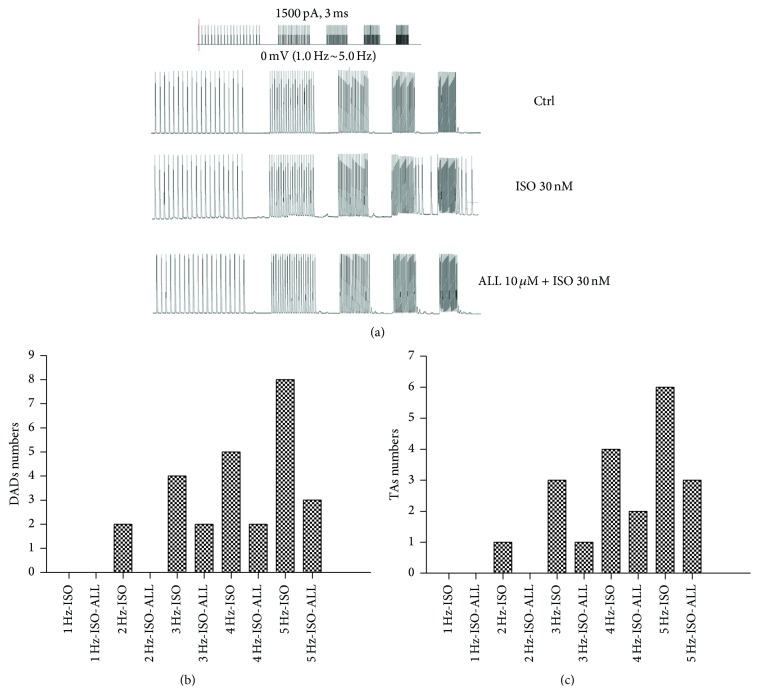
Frequency characteristics of ALL on DADs and TAs. (a) Hold potential at 0 mV, applying 30 wave pulses at 1500 pA for 3 ms at frequencies of 1.0, 2.0, 3.0, 4.0, and 5.0 Hz. Compared with untreated myocytes, the events of DADs and TAs progressively increased and had accelerated frequency following treatment with 30 nM ISO. Numbers of DADs or TAs were reduced in myocytes treated with ISO after exposure to 10 *μ*M ALL in (b and c).

Action potentials were recorded from ventricular myocytes that were stimulated at 1 to 5 Hz.  Figure 3(a) displays 30 continuous driven action potentials at each pacing frequency with the development of DADs and TAs when pacing was discontinued. DADs and TAs were observed after relatively faster pacing frequencies. Compared to control myocytes, the occurrence of DADs and TAs progressively increased following treatment with 30 nM ISO at higher frequencies. The numbers of DADs or TAs were reduced after exposure to 10 *μ*M ALL, and this effect was more significant during high frequency stimulation (Figures 3(b) and 3(c)).

### 3.3. *I*_ti_ of Mouse Ventricular Myocytes with ALL Treatment

**Figure 4:**
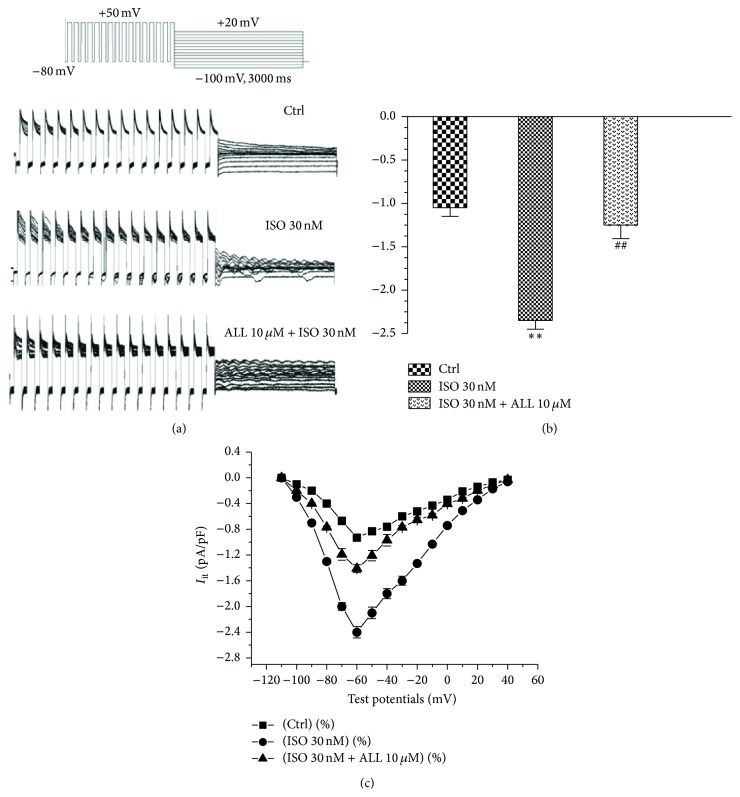
Effect of ALL on *I*_ti_ in mouse ventricular myocytes. (a) The left inset shows current traces obtained by applying train pulses from −100 mV to +30 mV for 2 s after conditioned stimulus ranging from −80 to +50 mV at intervals of 150 ms. (b) The larger current densities induced by ISO were significantly reduced with 10 *μ*M ALL treatment. (c) Current-voltage relationship showed that densities of inward *I*_ti_ ranging from −80 mV to 0 mV were decreased by treatment with ALL with the largest reduction of peak current at −60 mV. ^*∗∗*^ *p* < 0.01, versus control myocytes; ^##^ *p* < 0.01, versus myocytes with ISO treatment.

It is known that the occurrence of DADs is due to instigation by *I*_ti_. To evaluate the role of *I*_ti_ in development of DADs and TA, we recorded *I*_ti_ among three groups. Compared to control cells, there was a notable increase of *I*_ti_ found under exposure to 30 nM ISO. On average, the peak current densities of *I*_ti_ increased from −1.03 ± 0.12 pA/pF to −2.38 ± 0.08 pA/pF, which was markedly reduced by 10 *μ*M ALL to −1.21 ± 0.14 pA/pF (*p* < 0.05, *n* = 10, Figures 4(a) and 4(b)). Current-voltage relationship curves indicated that maximal inward current density of *I*_ti_ was at the potential of −60 mV. Also, the current density of *I*_ti_ was greater in myocytes with ISO treatment, ranging from −80 mV to −20 mV, compared to untreated myocytes. The ISO-induced increase of current in myocytes was reduced by ALL treatment (Figure 4(c)).

### 3.4. *I*_Ca,L_ of Mouse Ventricular Myocytes with ALL Treatment

**Figure 5:**
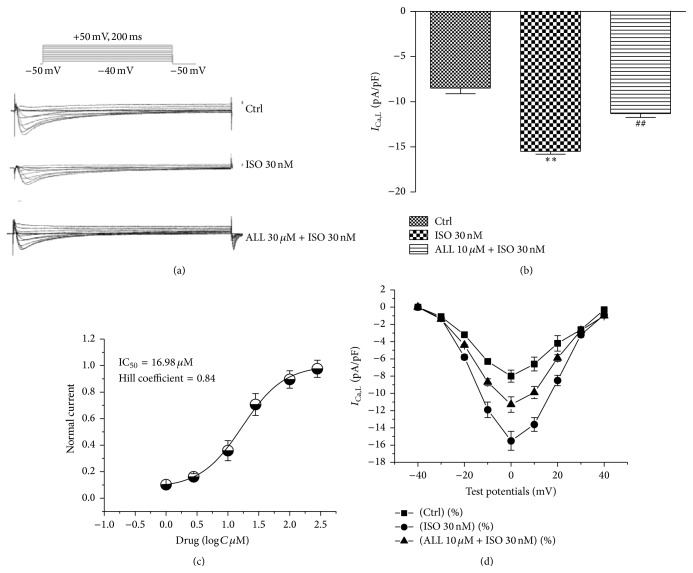
Effect of ALL on *I*_Ca,L_ in mouse ventricular myocytes. (a) Representative *I*_Ca,L_ traces in mouse ventricular myocytes of the indicated three groups. Current amplitudes of *I*_Ca,L_ with ISO treatment were significantly higher and were reduced by treatment with ALL (b). The concentration-dependent inhibition of ALL on *I*_Ca,L_ is shown in (c), with IC_50_: 16.08 *μ*M and Hill coefficient: 0.84. The current-voltage relationship demonstrated that current densities of *I*_Ca,L_ from myocytes with ISO treatment were significantly larger than in control myocytes. The effect of ISO on *I*_Ca,L_ was alleviated by 10 *μ*M ALL treatment (d). ^*∗∗*^ *p* < 0.01, versus control myocytes; ^##^ *p* < 0.01, versus myocytes with ISO treatment.

Since DAD events were augmented by both ISO and ALL treatment, we mainly measured currents of L-type calcium currents. The *I*_Ca,L_ currents of three groups were showed in Figure 5(a). The current densities of *I*_Ca,L_ in myocytes with 30 nM ISO treatment were significantly larger, with −8.5 ± 0.6 pA/pF in the control group and −15.5 ± 0.3 pA/pF in the ISO-treated group at 0 mV of test potential. *I*_Ca,L_ was reduced to −10.3 ± 0.4 pA/pF in ISO-treated myocytes that were cotreated with 10 *μ*M of ALL (*p* < 0.01, *n* = 12, Figure 5(b)). The concentration-dependent inhibition of ALL on *I*_Ca,L_ is shown in Figure 5(c), with IC_50_: 16.08 ± 1.23 *μ*M and Hill coefficient: 0.84. The current-voltage relationship demonstrated that current densities of *I*_Ca,L_ from myocytes treated with ISO were significantly larger than in control myocytes from −20 mV to +20 mV. This effect was alleviated by 10 *μ*M ALL treatment (Figure 5(d)).

**Figure 6:**
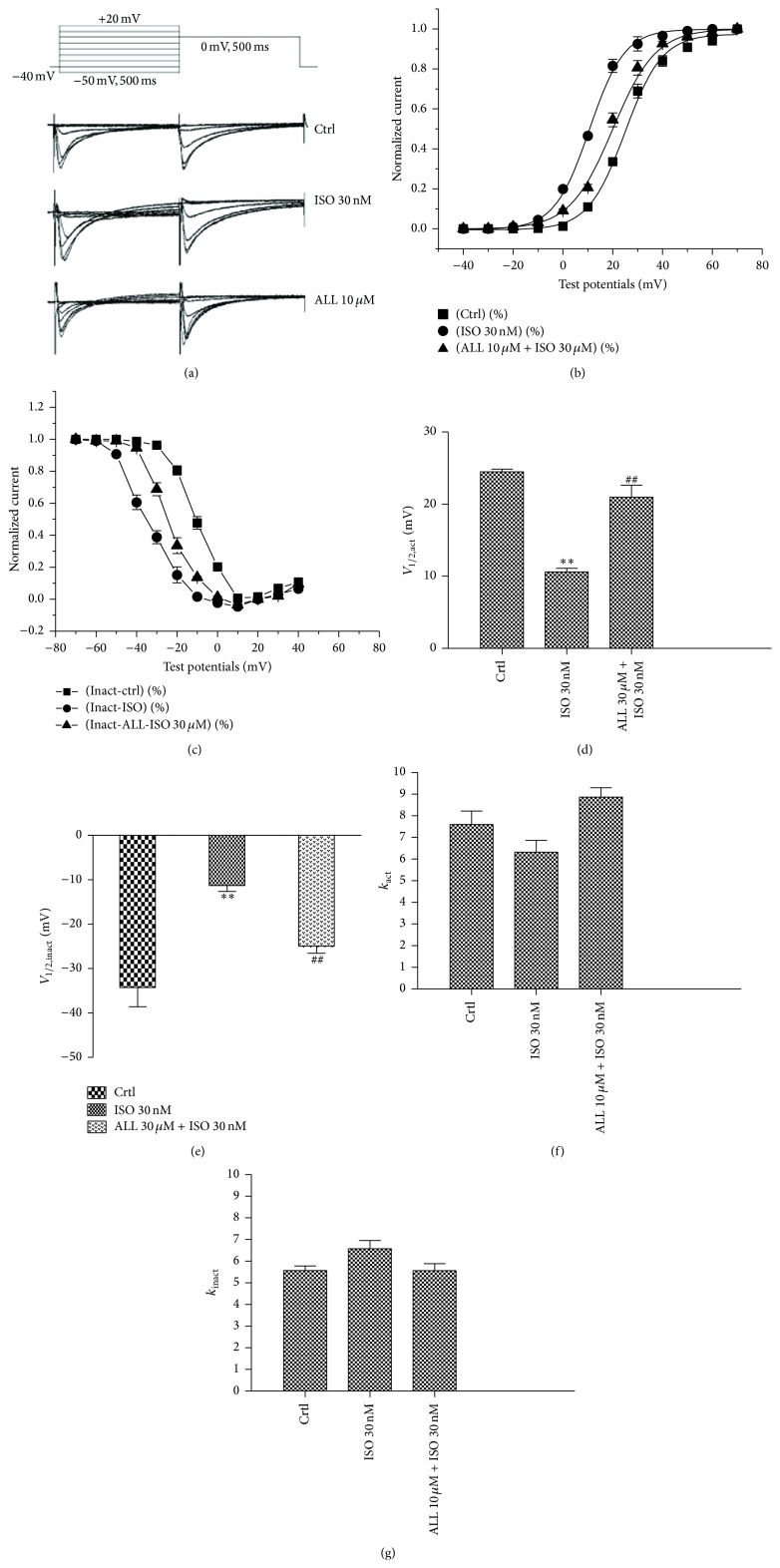
Effect of ALL on steady-state activated and inactivated curve of *I*_Ca,L_. (a) The representative currents of *I*_Ca,L_ activation and inactivation before and after being treated with Iso 30 nM and ALL 10 *μ*M. (b and c) The steady-state activated curve of *I*_Ca,L_ was shifted to negative potential, while the steady-state inactivated curve was shifted to positive potential following ISO treatment. Changes of steady-state (in)activated curves were reversed with 10 *μ*M ALL treatment. *V*_1/2,act_ and *V*_1/2,inact_ were shown in (d and e); *k*_act_ and *k*_inact_ were shown in (f and g). ^*∗∗*^ *p* < 0.01, versus control myocytes; ^##^ *p* < 0.01, versus myocytes with ISO treatment.

Steady-state activated curves and steady-state inactivated curves of *I*_Ca,L_ were fitted by the Boltzmann equation function. The steady-state activated curve of *I*_Ca,L_ was shifted to more negative potential in the presence of 30 nM ISO. Meanwhile, the steady-state inactivated curve was shifted to more positive potential by ISO treatment (Figures 6(a) and 6(c)). This suggests a slower activated procedure and faster inactivated procedure of *I*_Ca,L_. Changes to the steady-state (in)activated curves were reversed by treatment with 10 *μ*M ALL. *V*_1/2,act_ and *V*_1/2,inact_ were significantly different between the three groups, whereas *k*_act_ and *k*_inact_ values in the three groups had no significant difference (Figures 6(b) and 6(d)).

**Figure 7:**
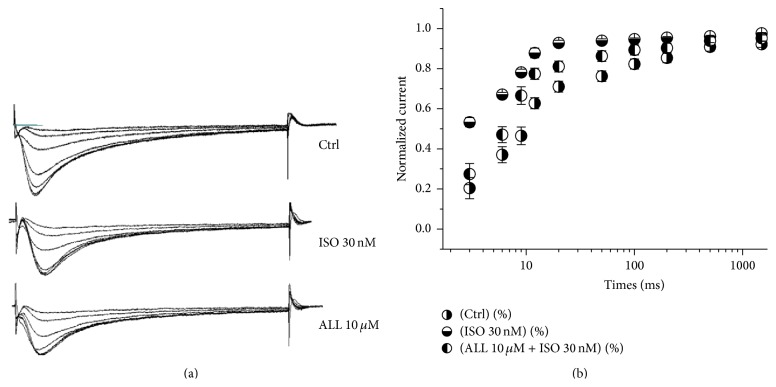
Effect of ALL on recovery from inactivation of *I*_Ca,L_. (a) The representative currents of *I*_Ca,L_ recovery from inactivation after being treated with Iso 30 nM and ALL 10 *μ*M. (b) A faster recovery from inactivation of *I*_Ca,L_ in myocytes treated with Iso was observed. The time constant of recovery from inactivation was prolonged in myocytes treated with ALL.

A faster recovery from inactivation of *I*_Ca,L_ in myocytes with ISO treatment was reversed by 10 *μ*M ALL treatment. The representative currents of recovery from inactivation before and after Iso and ALL treatment were recorded and shown in Figure 7(a). The average recovery time constants from inactivation were 895 ± 20 ms in the control group, 312 ± 16 ms in the ISO-treated myocytes, and 545 ± 22 ms in myocytes cotreated with ISO and ALL, respectively (*p* < 0.01, *n* = 10, Figure 7(b)).

## 4. Discussion

The major finding of the present study is that mouse ventricular myocytes exhibited a lower incidence of ISO-induced DADs and TAs in response to 10 *μ*M ALL, which suggests that ALL has potential antitriggered arrhythmic effects. TAs have been easily induced by DADs in ventricular myocytes following the addition of isoproterenol and high calcium [8]. Furthermore, in isolated mouse ventricular myocytes, the effect of ALL on depressing DADs and DAD-induced TAs is largely rate-dependent showing stronger inhibitory effects at higher pulse frequencies.

DADs are generally thought to be initiated by spontaneous Ca^2+^ release from the sarcoplasmic reticulum (SR) and a Ca^2+^-activated transient, depolarizing inward current (*I*_ti_) [9, 10]. Several Ca^2+^-activated currents have been proposed to participate in *I*_ti_, namely, the NCX exchange (*I*_Na/Ca_) and calcium activated chloride channels (*I*_Cl,Ca_) [11, 12]. Recently, Asakura et al. [4] reported that activation of the ryanodine receptor results in an explicit Ca^2+^ release initiated by the subthreshold Ca^2+^ accumulation and the progressive accumulation of [Ca^2+^]_i_, which rapidly increases the rate of DAD events. During heart failure, it has been shown that there is an increase in magnitude of *I*_Na_ and *I*_Ca,L_ and NCX exchange protein can double. Together, this will increase *I*_Na/Ca_ and will double *I*_ti_ amplitude, which depolarizes the cell towards TA threshold. *I*_ti_ induces DADs during the diastolic interval that may trigger premature beats by reaching threshold potential. Triggered arrhythmias cause the majority of sudden cardiac death during nonischemic heart failure [2]. Our data demonstrates the ability of ALL to suppress DADs and DAD-induced TA and that this effect could be explained by its action to reduce *I*_ti_ currents. Current-voltage relationships of myocytes undergoing stimulation showed that densities of inward *I*_ti_ range from −80 mV to 0 mV were decreased by ALL, with the largest reduction peak current at −60 mV. The lower amplitude of *I*_ti_ leads to fewer events of DADs and subsequent DAD-induced TAs.

During heart failure, *I*_Ca,L_ is essential for the electrical and biochemical function of the heart because they are the primary pathway for Ca^2+^ influx into cardiac myocytes. We found a notable *I*_Ca,L_ increase in myocytes with ISO treatment. The steady-state activated curve of *I*_Ca,L_ was shifted to negative potential, while the steady-state inactivated curve was shifted to positive potential by ISO treatment. The main mechanism of channel gating is a shorter time constant of recovery from inactivation in ISO-treated cells. The time constant of recovery from inactivation was prolonged and changes of the steady-state (in)activated curves were reversed by ALL treatment. Also, the increased effect of ISO on *I*_Ca,L_ was alleviated by ALL with less DAD events as a response. For these reasons and more, the effects of ALL on *I*_Ca,L_ and *I*_ti_ may attenuate ISO-induced arrhythmogenesis by decreasing the genesis of DADs.

The data in this study should be interpreted with caution because of its potential limitations. First, normal mouse ventricular myocytes are employed to determine the effect of ALL on DADs and TAs. As we know, triggered arrhythmias are prone to develop in heart failure. It is difficult to establish a heart failure model in mice, but normal cells do not mirror disease states. Therefore, ventricular myocytes isolated from mice with heart failure should be adopted in future research. Second, DADs and TA events are regulated by many currents such as *I*_Ca,L_, *I*_ti_, *I*_K1_, *I*_NCX_, and *I*_Cl,Ca_ [13, 14]. We only investigated *I*_Ca,L_ and *I*_ti_ in this study. The effects of ALL on other currents should be investigated in future research.

In conclusion, DADs and TA events were decreased in mice ventricular cells treated with ALL. The underlying mechanism through which these cells exhibited a lower incidence of triggered arrhythmias was unclear. A possible explanation is that the decrease of *I*_Ca,L_ leads to NCX exchanger inhibition and generates *I*_ti_. On the other hand, *I*_ti_ current can be directly blocked by ALL. Together, these effects on *I*_ti_ will explain the reduced incidence of DADs and TAs in mouse by ALL.
